# ATM in senescence

**DOI:** 10.18632/oncotarget.4411

**Published:** 2015-06-10

**Authors:** Katherine M. Aird, Rugang Zhang

**Affiliations:** Gene Expression and Regulation, The Wistar Institute, Philadelphia, PA, USA

Senescence is a state of stable cell growth arrest that can be triggered by multiple stressors, including oncogene activation [1]. In normal diploid mammalian cells, activation of oncogenes such as oncogenic RAS or BRAF decreases dNTP levels, which leads to replication stress and ultimately senescence [2]. In this context, oncogene-induced senescence (OIS) is considered to be an important tumor suppressor mechanism [1]. Overcoming OIS is necessary for cell transformation, which may ultimately lead to cancer development [3]. Therefore, understanding the basic mechanisms whereby cells bypass OIS is important for understanding the earliest events in tumorigenesis. Additionally, knowledge of the pathways that can overcome replication stress-induced senescence may allow for targeting of these specific pathways for cancer therapy or prevention.

We previously found that during OIS, downregulation of ribonucleotide reductase M2 (RRM2), the rate-limiting enzyme in dNTP biosynthesis, underlies the observed replication stress [2]. This correlates with a significant decrease in dNTP levels and the associated DNA damage response (DDR). This leads to the establishment and maintenance of the stable senescence-associated cell growth arrest observed during OIS. Ectopic RRM2 or supplementation of cells with exogenous nucleosides is sufficient to overcome OIS, demonstrating the importance of nucleotide metabolism to OIS and the associated stable cell growth arrest.

A question that arises from our previous study is how can cells overcome replication stress-induced senescence? To address this important question, we examined the effects of inhibition of ATM and ATR, two major players in the DDR that are activated during replication stress induced by RRM2 suppression [4]. Notably, inactivation of ATM, but not ATR, was able to overcome senescence induced by replication stress. Consistently, loss or mutations in ATM, but not ATR, increases cancer risk [5]. Senescence bypass induced by ATM inactivation correlates with an increase in cellular dNTP levels, further highlighting the importance of dNTP levels in the replication stress observed during senescence. This was neither due to compensation by other nucleotide metabolic pathway enzymes nor increased dNTP salvage. Instead, restoration of cellular dNTP levels by ATM inactivation in the context of replication stress is accompanied by metabolic reprogramming.

**Figure 1:**
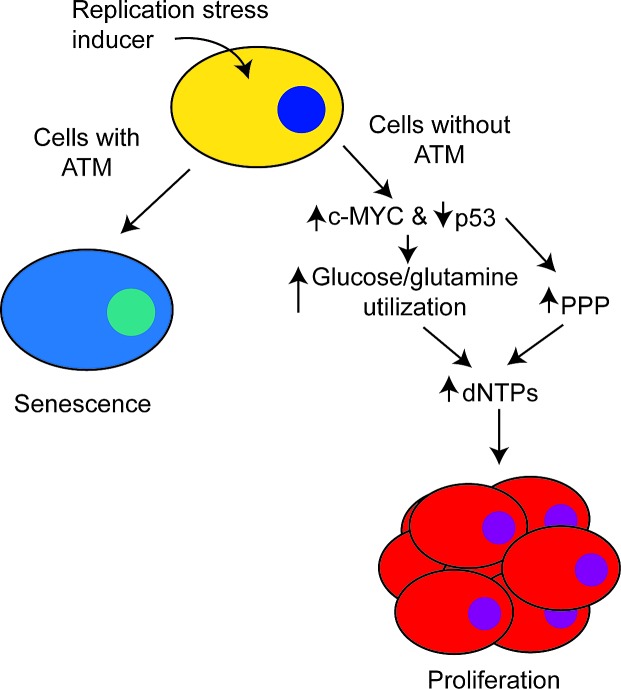
ATM inactivation overcomes replication stress-induced senescence In cells with functional ATM, replication stress induces senescence. In cells without ATM, there is a coordinated upregulation of c-MYC and downregulation of p53 to increase glucose and glutamine utilization. Additionally, decreased p53 increases activity of the pentose phosphate pathway (PPP). This reprogramming in cellular metabolism increases dNTPs, which allows for cells to overcome the senescence-associated cell growth arrest to proliferate.

It has recently become clear that changes in metabolism occur during OIS, which often oppose the cancer-associated Warburg Effect [6]. We found that in the context of replication stress, loss of ATM increased glucose and glutamine consumption and utilization, similar to what is observed in cancer cells [3]. This suggests that reprogramming of cellular metabolism is sufficient to switch cells from a tumor suppressive to a tumor promoting phenotype. The observed metabolic reprogramming induced by ATM inactivation occurs through a coordinated downregulation of p53 activity and upregulation of c-MYC stability. Wild-type p53, but not its cancer-associated mutants, is known to suppress the activity of glucose-6-phosphate dehydrogenase (G6PD), the rate-limiting enzyme in the pentose phosphate pathway (PPP). The PPP is necessary for production of ribose-5-phosphate, which is the sugar base of all nucleosides. Consistently, ATM inactivation increased G6PD activity in a p53 mutational status-dependent manner. Therefore, ATM inactivation suppresses replication stress and the associated senescence by restoring cellular dNTP levels through a coordinated increase in nutrient substrates and activity of the PPP for dNTP biosynthesis (Figure 1).

Our results demonstrate that in addition to its classical tumor suppressive role in the DDR, ATM functions as a tumor suppressor by suppressing cancer-associated metabolism to promote senescence and the associated stable cell growth arrest. In our report, we found that inactivation of ATM coordinately suppressed p53 and activated c-MYC. Analysis of The Cancer Genome Atlas (TCGA) datasets indicates a mutual exclusivity between ATM inactivation and p53 mutation/inactivation/c-MYC amplification. This further supports our central hypothesis that these alterations function in the same pathway (where ATM is upstream of p53 and c-MYC) and either ATM inactivation or p53 inactivation together with c-MYC upregulation can induce the observed metabolic reprogramming.

Our results have potential implications for developing cancer therapeutic strategies. There has been continuous interest in developing ATM inhibitors for utilization as a cancer therapy. However, based on our study, inhibition of ATM may lead to metabolic reprogramming that could enhance the cancer-associated Warburg Effect. This will need to be evaluated with caution to determine the impact on long-term changes in the malignant behavior of cancer cells given that metabolic pathways affect many aspects of cancer biology. Additionally, it is possible that patients with inactivated ATM will be more sensitive to therapeutic intervention using metabolic inhibitors. Further studies are warranted to determine the metabolic weaknesses exposed by ATM inactivation in order to target them with metabolic inhibitors.

In conclusion, we found that ATM inactivation reprograms cellular metabolism to overcome replication stress-induced senescence. Understanding how cells overcome the tumor suppressive metabolism observed during senescence may lead to ways to prevent transformation of these cells or new ways to target these pathways in order to develop new cancer therapeutic strategies.
